# Improvement in cardiometabolic risk markers following a multifunctional diet is associated with gut microbial taxa in healthy overweight and obese subjects

**DOI:** 10.1007/s00394-017-1563-3

**Published:** 2017-11-02

**Authors:** Nittaya Marungruang, Juscelino Tovar, Inger Björck, Frida Fåk Hållenius

**Affiliations:** 1 Department of Food Technology, Engineering and Nutrition, Lund University, Kemicentrum, Sölvegatan 39, SE-22100 Lund, Sweden; 2 InnovaFood AB, Äppelvägen 15, 247 47 Flyinge, Sweden

**Keywords:** Cardiovascular, Microbiota, Diet, Obese, Human, Prevention

## Abstract

**Purpose:**

A multifunctional diet (MFD) targeting subclinical inflammation was developed as a tool to decrease risk factors for cardiometabolic disease in healthy “at-risk” individuals (BMI 25–33 kg/m^2^). MFD contains several components that are degraded in the colon by the microbiota, such as dietary fibers from rye, barley, oats and berries. It also contains soy beans, oily fish and plant stanols. In previous studies, we have observed improved cardiometabolic markers in healthy at-risk individuals after 4–8 week intake of MFD. However, whether these improvements can be associated with changes in the gut microbiota composition has not been investigated. In the present study, we analyzed the gut microbiota before and after an 8-week dietary intervention with MFD.

**Methods:**

Cardiometabolic at-risk individuals (*n* = 47), between 51 and 72 years old and with a BMI of 25–33 kg/m^2^, were given either the MFD or a control diet lacking the functional (“active”) components for 8 weeks in a parallel, randomized design. Next-generation sequencing of bacterial 16S rRNA genes was used to analyze the gut microbiota composition.

**Results:**

The 8-week intervention with MFD did not significantly alter the gut microbiota composition at phylum or genus taxonomic levels, while LEfSE analysis identified increased abundance of *Prevotella copri* in the MFD group as compared to the control group. *Treponema* correlated positively with blood pressure. In contrast, *Faecalibacterium* showed a negative association with blood pressure, while *Bilophila* appeared to associate with a negative blood lipid profile.

**Conclusions:**

Taken together, results from the present study may be used in the further development of effective dietary concepts capable of reducing cardiometabolic risk markers in humans through a targeted modulation of the gut microbial community.

**Trial registration number:**

Clinical Trials.gov NCT02148653.

**Electronic supplementary material:**

The online version of this article (doi:10.1007/s00394-017-1563-3) contains supplementary material, which is available to authorized users.

## Introduction

Ingested food components, preferentially carbohydrates that are not digested and absorbed in the small intestine, reach the colon where they serve as fuel for the inherent microbiota. Hence, the diet is of utmost importance in shaping the composition of the gut microbiota. In turn, the gut microbiota affects the host’s energy metabolism, glucose homeostasis and inflammatory status to a large extent [1–3]. Recent work by Klinder et al. using fluorescence in situ hybridization (FISH) showed that increased intake of fruits and vegetables altered the fecal microbiota in humans, with, e.g., increased levels of *Ruminococcus bromii* and increased *Bacteroides*/*Prevotella* ratio, as well as indicated associations between cardiovascular risk markers and specific bacteria [4]. Other dietary components, such as walnuts and pomegranate, can also be metabolized by the gut microbiota. These metabolites, urolithins, can be detected in blood and used as markers for cardiometabolic risk in humans [5]. Other biomarkers more commonly used are c-reactive protein (CRP), cytokines and blood lipids. However, low-grade inflammation observed in individuals with the metabolic syndrome is a complex physiological state that may require analysis of a cluster of biomarkers, as reviewed by Minihane et al. [6]. New markers for cardiometabolic risk are essential to investigate further, as they are key in developing prevention strategies against cardiovascular disease. A multifunctional diet (MFD) targeting subclinical inflammation was developed as a tool to decrease risk factors for cardiometabolic disease in at-risk individuals, i.e., in mature individuals (> 51 years of age) with overweight or obesity. The diet includes foods and/or meals with anti-inflammatory potential in that they promote low acute glycemic responses, are rich in polyphenols and/or specific dietary fiber with prebiotic action, are rich in omega 3 fatty acids such as oily fish and rapeseed oil, or with anti-oxidative and anti-hypercholesterolaemic effects, e.g., soybeans and almonds [7].

MFD contains several components that are degraded in the colon by the microbiota, such as dietary fibers from rye, barley, oats and berries. In previous studies, we have observed improved cardiometabolic markers in healthy at-risk individuals after 4–8 week intake of MFD targeting low-grade inflammation [7, 8]. The diet exerted also extensive impact on the plasma metabolome, particularly the lipidome [9]. However, whether these improvements can be associated with changes in the gut microbiota composition has not been investigated. In the present study, we analyzed the gut microbiota before and after the 8w dietary intervention with MFD in subjects from [8] using next-generation sequencing of bacterial 16S rRNA genes. At-risk individuals were given either the MFD or a control diet lacking the functional (“active”) components for 8 weeks in a parallel, randomized design. Fecal samples were collected at baseline and at the end of each study arm for microbiota analysis. We could identify bacterial taxa that were associated with the MFD and, correspondingly, with improved cardiometabolic risk markers.

## Materials and methods

### Dietary intervention

#### Study protocol

The dietary intervention is described in detail by Tovar et al. [8]. Briefly, the study was designed as a randomized, controlled, parallel 8-week trial of the effect of MFD on biomarkers related to cardiometabolic risk. The participants were randomly assigned, using a computerised random number generator, to one of two treatments; MFD or control diet (CD), respectively. Primary endpoint was the change in fasting plasma LDL cholesterol. Assuming a 0.7 mmol/L (approximately 20%) post-diet difference and a 0.99 SD, with *α* = 0.05 and 1 − *β* = 0.8, a minimum of 44 participants (22 per treatment) were required.

Both at the beginning and end of the trial, fasting body weight and blood pressure (BP) were recorded. Venous blood was drawn both at the start and end of the intervention for the assessment of fasting glucose, insulin, glycated hemoglobin, cholesterol (total, low density lipoprotein (LDL) and high-density lipoprotein (HDL)), triacylglycerols (TAG), high-sensitivity C-reactive protein (hs-CRP), apolipoprotein A1 (apoA1), apolipoprotein B (apoB), interleukin 6 (IL-6), tumor necrosis factor alpha (TNF-α), free fatty acids and plasminogen activator inhibitor 1 (PAI-1) levels. Fecal samples were also collected on the day before the start of the trial and during the last day of the intervention and kept at − 40 °C until analyzed.

To ensure compliance, the participants were provided with a detailed 2-week rotating menu plan for each dietary period (MFD and CD) with all ingredients expressed in weight and/or volume measures. The menu plan also included recipes for preparing meals. Compliance was calculated from the intake scores for the functional components in MFD (soybean and soy protein-based products, barley, rye, blue berries, cinnamon, plant stanol-containing margarine, fish, vinegar, rapeseed oil, almond, whey protein, guar gum), and relevant dietary fibre-providing items in CD (processed cereals, white wheat bread, dark wheat bread, fruits and vegetables).

#### Participants

Non-smoking healthy volunteers, i.e., over-weight or obese but without any known medical condition, were recruited through advertisement in local newspapers and were informed orally and in writing of practical details of the experiment. Aiming to address a healthy cohort with increased risk for developing cardiometabolic complications, the inclusion criteria were: age between 50 and 73 years old, body mass index (BMI) in the 25–33 kg/m^2^ range and fasting plasma glucose value ≤ 6.1 mmol/L.

Subjects were recruited between February and March 2014 and the clinical visits took place between March and June 2014. The volunteers came from towns and villages in Southern Sweden. A total of 52 subjects were recruited and 51 subjects were enrolled in the study. Twenty-three subjects completed the multifunctional diet arm and 24 completed the control diet intervention. Thus, results from 47 completers (12 men and 35 women) were analyzed.

#### Diets

The nutritional profiles of CD and MFD were described previously. Both diets were designed in agreement with the Nordic Nutrition Recommendations [10] and supplied 2500–2600 Kcal/day for men and 2000–2100 Kcal/day for women, combining foods from plant and animal origins. Both diets incorporated commercial foods available in food stores, but the MFD also included prototype products. For a detailed list of products included in MFD, see [7]. MFD combined several functional concepts with potential ability to modulate different biomarkers related to the inflammatory tonus and cardiometabolic risk, including:

(a) natural antioxidant-rich foods which, in addition to the anti-inflammatory action of their antioxidants, contain phenolics that may improve blood pressure and blood lipids; (b) omega-3 fatty acids, especially those long-chained present in oily fish, which have anti-inflammatory and triglyceride-lowering properties. In addition, the overall fat quality was also a concept included in the diet design. The source of omega-3 fatty acids was from oily fish and rapeseed oil. Thus, the unsaturated-to-saturated fat ratio was larger in MFD than in CD (3.6 vs 1.9, respectively); (c) ingredients with prebiotic activity, i.e., non-starch polysaccharides and resistant starch in intact barley kernels, whole kernel rye flour and isolated fiber, i.e., beta-glucans. Additional sources of viscous fermentable dietary fibre were a prototype oat-based fiber drink, a rye/oat breakfast cereal, an oat-based muesli and an experimental guar gum-containing bread; (d) low glycemic impact foods/meals were included for their association with reduced risk for cardiometabolic problems and their perceived ability to ameliorate the inflammatory tonus in healthy individuals. Additionally, MFD included ingredients that lower the postprandial glycemic response, such as whey protein and vinegar; (e) blood cholesterol-normalizing ingredients: different soybean products, a margarine enriched in stanol esters and dry almonds.

None of the active ingredients were included in the CD, except for minor amounts of ω-3 fatty acids. MFD provided 2.0 g stanols/day for women and 2.7 g/day for males. The total dietary fibre content was 24 g/day for individuals consuming the CD and 62 g/day for the MFD. The dietary fiber in the CD was mainly from fruits, vegetables and wheat, with little contribution from wholegrain products. Representative 1-day menus can be found in the supplementary material.

### Gut microbiota composition

#### DNA extraction

Faecal samples were thawed on ice and DNA was extracted using the QIA amp DNA Stool Mini Kit (Qiagen). The protocol from the manufacturer was followed with an addition of a bead beating step. Sterile glass beads (1 mm) were added together with stool lysis buffer to the samples and cell disruption was performed for 2 × 2 min at 25 Hz using a TissueLyser (Qiagen), followed by a heating step at 95 °C for 5 min. After lysis, DNA-damaging substances and PCR inhibitors were removed using InhibitEX tablet (provided with the kit) and the DNA was purified on QIAamp Mini spin columns.

#### PCR amplification of the V3-4 region of bacterial 16S rRNA genes

16S rRNA genes were amplified by PCR with forward and reverse primers containing Illumina adapter sequences and unique dual indexes used to tag each PCR product, according to the 16S-protocol provided by Illumina. Primer sequences can be found in Table 1. Briefly, PCRs were carried out in 25-μL reactions with 0.2 μM forward and reverse primers, with 12.5 ng template DNA and 12.5 µl of 2 × KAPA HiFi HotStart Ready Mix kit (KAPA Biosystems). Thermal cycling consisted of initial denaturation at 95 °C for 3 min followed by 25 cycles of denaturation at 95 °C for 30 s, annealing at 55 °C for 30 s, and extension at 72 °C for 30 s, followed by a final step of 72 °C for 5 min. The amplicons (550 bp product) were purified with Agencourt AMPureXP Kit (Beckman Coulter). A second PCR was thereafter performed to attach Illumina adapters and unique dual indexes to each sample, followed by a clean-up step with AmPureXP Kit (Beckman Coulter). PCR amplicons were visualized using 0.1% agarose gel electrophoresis. Negative extraction controls did not produce visible bands.

**Table 1:** Primer sequences for amplification of 16S rRNA genes, amplicon length 550 bp

16S Amplicon PCR Forward Primer with Illumina overhang adaptor (underlined)	5′ TCGTCGGCAGCGTCAGATGTGTATAAGAGACAGCCTACGGGNGGCWGCAG
16S Amplicon PCR Reverse Primer with Illumina overhang adaptor (underlined)	5′ GTCTCGTGGGCTCGGAGATGTGTATAAGAGACAGGACTACHVGGGTATCTAATCC

#### Amplicons quantitation, pooling, and sequencing

Faecal amplicon DNA concentrations were quantified using the Qubit3.0 Fluorometer (Life Technologies, Stockholm, Sweden). Amplicons were combined in equimolar ratios into a single tube with a final concentration of 4pM. As an internal control, 5% of PhiX was added to the amplicon pool. Paired-end sequencing with a read length of 2 × 300 bp was carried out on a Miseq Instrument (Illumina) using a Miseq reagent kit v3 (Illumina Inc., San Diego, USA).

#### Sequence analysis

Sequences were analyzed with the free software package Quantitative Insights into Microbial Ecology (QIIME) which allows analysis of high-throughput community sequencing data. Default values were used for each step, except where otherwise specified [11]. Sequences were removed if lengths were < 200 nucleotides, contained ambiguous bases, primer mismatches or homo polymer runs in excess of six bases. Forward and reverse reads were joined using Fastqjoin. After quality filtering, a total of 11,375,395 sequence reads were generated for the dataset with an average number of 121,014 reads per sample. Similar sequences were binned into operational taxonomic units (OTUs) using UCLUST [12], with a minimum pairwise identity of 97%, generating 5,756 OTUs, using the closed-reference OTU picking method in QIIME. The most abundant sequence in each OTU was chosen to represent its OTU. Representative sequences from each OTU were aligned using PyNAST (a python-based implementation of NAST in QIIME) and taxonomy was assigned using the Greengenes database (v. 13_8) [13] and the RDP classifier [14]. Also de novo OTU picking in QIIME was applied on the dataset to identify novel species of bacteria.

### Statistical analysis

GraphPad Prism 6 (GraphPad software, Inc., La Jolla, California) was used to identify significant differences in bacterial relative abundances in faeces following the MFD and CD, respectively, using multiple t tests and the Holm–Sidak method of correction for multiple comparisons, at phylum and genus level. To find correlations between cardiometabolic risk markers and bacterial genera, the operational taxonomic units (OTU) tables were rarefied at 50,620 randomly selected sequences/sample, for the entire data set after which partial least squares (PLS) scatter plot analysis was performed using SIMCA14 (Umetrics, Umeå, Sweden). Pearson’s correlation was calculated for each pairwise combination of risk markers and bacterial genera using Minitab17 (Minitab Inc, State College, Pennsylvania) and the p values were then corrected using Benjamini–Hochberg procedure for multiple comparisons [15, 16]. Further, α- and β-diversities were analyzed in QIIME on rarefied data, using 50,620 sequences/sample. Differences in α-diversity were analyzed in QIIME using a t test and FDR correction for multiple comparisons and β-diversity differences were analyzed with the ANOSIM and Adonis non-parametric statistical tests in QIIME. To identify bacterial species that could be identified as biomarkers related to each diet, linear discriminant analysis effect size (LEfSE, https://huttenhower.sph.harvard.edu/galaxy/) was applied on the OTU table according to Segata [17]. The functional capacity of the gut microbiome was estimated by inferring metabolic functionality from the 16S rRNA gene sequencing data using an open-source software, Phylogenetic Investigation of Communities by Reconstruction of Unobserved States (PICRUSt) [18].

## Results

### The multifunctional diet improved cardiometabolic risk markers

As described previously, the 8-week intervention with MFD improved a range of cardiometabolic risk markers in healthy mature and overweight subjects, while the CD did not have significant metabolic effects [8]. Both the MFD and the CD resulted in a significant weight loss from baseline, where the MFD group changed from 78.6 ± 0.9 (baseline) to 75.8 ± 1.8 kg (after 8 weeks, *P* < 0.01 compared to baseline), and the CD group changed from 79.3 ± 2.1 to 76.3 ± 2.1 kg (*P* < 0.01 compared to baseline). Weight changes did not differ between diets (*P* = 0.481). Besides slightly decreased body weight and diastolic blood pressure, consumption of MFD promoted marked reductions in total circulating cholesterol (− 26%, *P* < 0.0001), LDL cholesterol (− 34%, *P* < 0.0001), LDL-to-HDL and Apo B-to-Apo A1 ratios (− 27%, *P* < 0.0001 and − 15% *P* < 0.0001, respectively) and triglycerides (− 16%, *P* < 0.05) compared to baseline [8]. Furthermore, the MFD reduced the Reynold’s cardiovascular risk score with 36% (*P* < 0.05), while the CD did not alter this risk score [8]. As a measure of gut fermentation, breath hydrogen was also measured, and was found to be increased by 120% in the MFD group compared to baseline (*P* < 0.05). Breath hydrogen instead decreased in the CD group by 32% (*P* < 0.05) [8]. The fermentation results indicated an altered gut microbiota composition and/or activity. Thus, in the present study, we performed a comprehensive next-generation sequencing of bacterial 16S rRNA genes from fecal samples harvested from the 8-week dietary intervention.

### The MFD induced only minor shifts in the gut microbiota composition

**Fig. 1:**
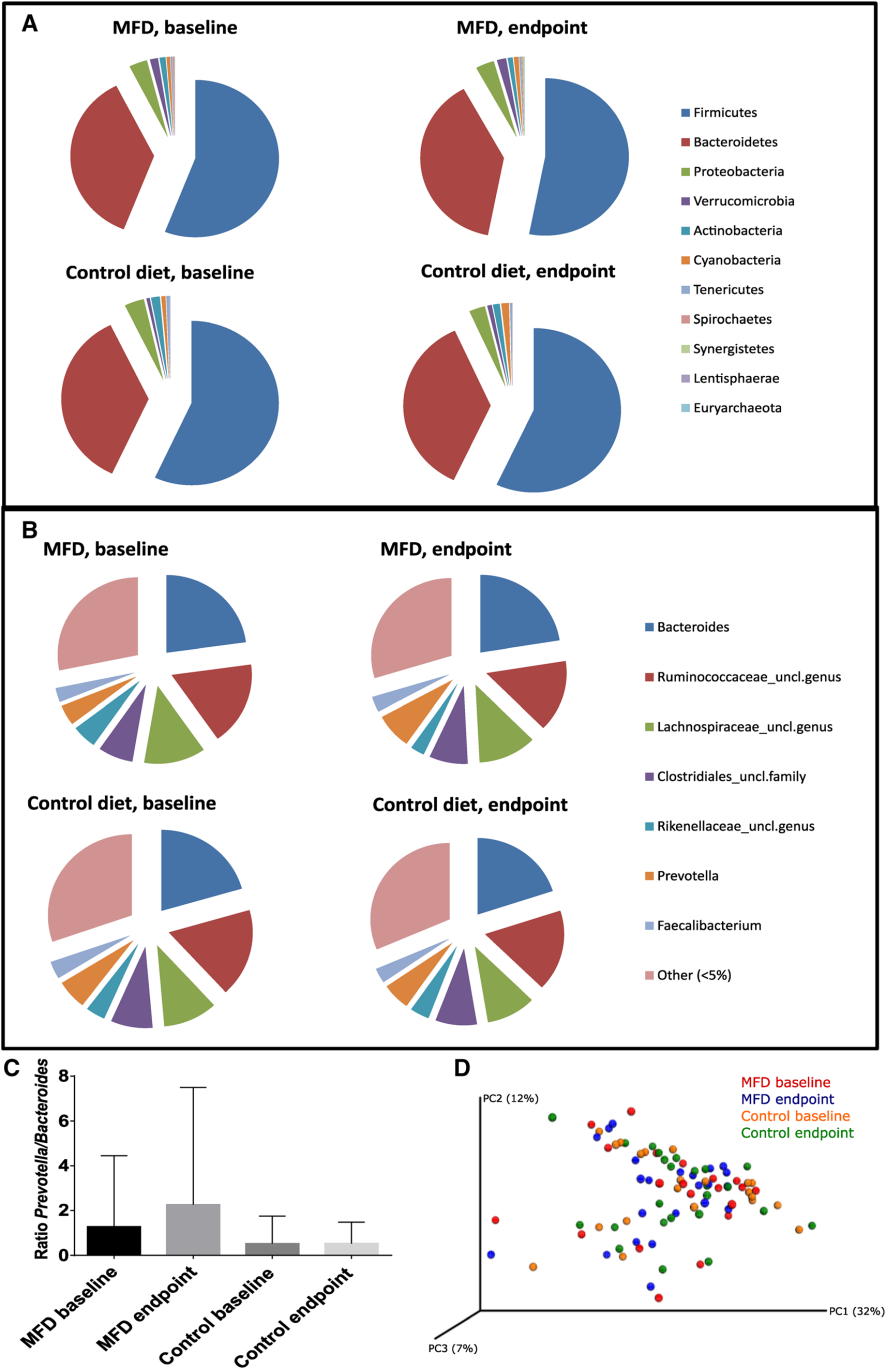
Gut microbiota composition in MFD and control groups at baseline and end point. Relative abundance of the gut microbiota at **a** phylum and **b** genus level. **c** *Prevotella*/*Bacteroides* ratio. **d** Weighted UniFrac PCoA plot showing gut microbial community composition among the groups at 50,620 randomly selected sequences/sample

**Fig. 2:**
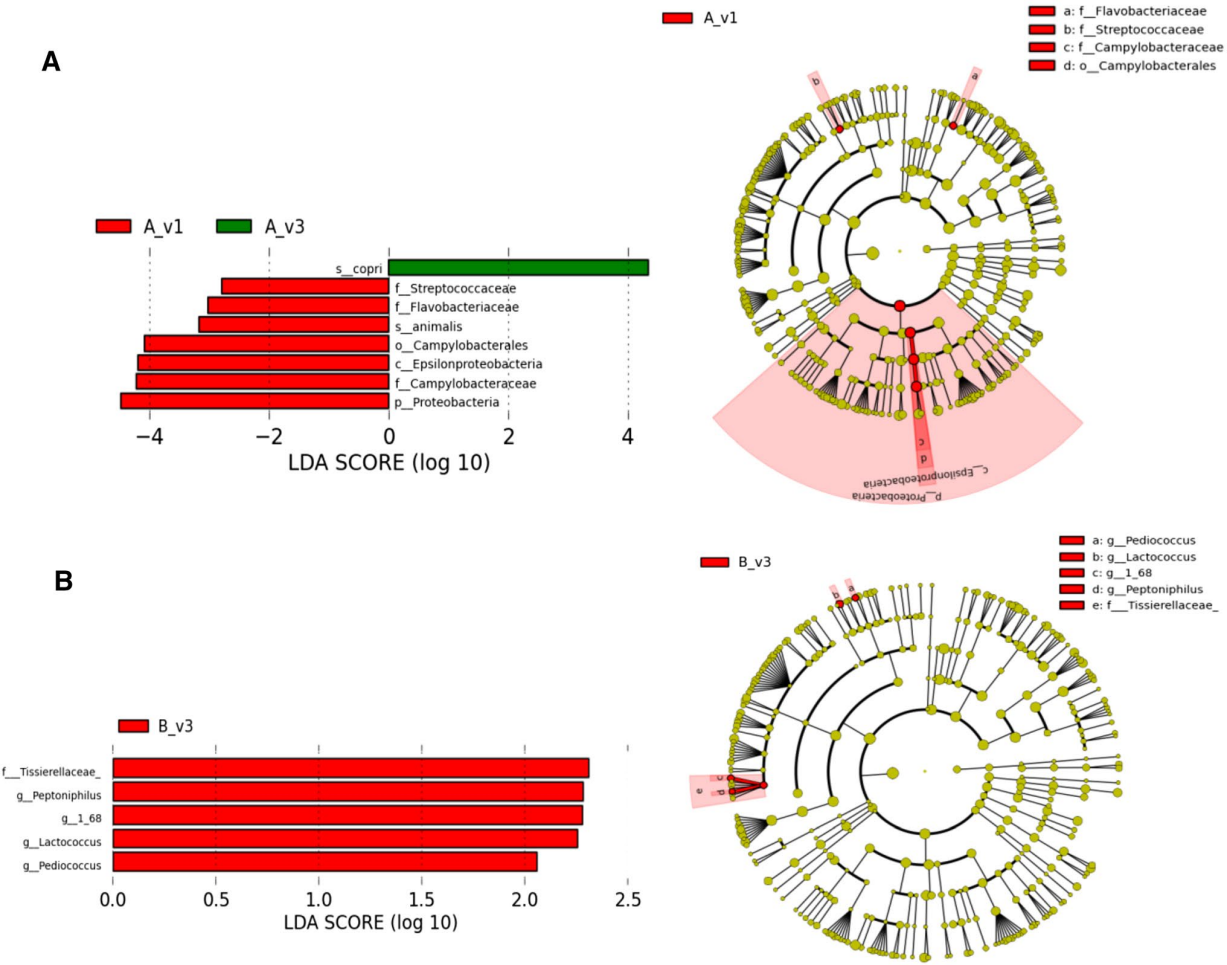
LDA score plot (left) and cladogram plot (right) from LEfSE analysis of the gut microbiota composition in MFD and control groups at baseline and end point. A = MFD, B = Control diet, v1 = baseline, v3 = end point. Microbial taxa shown have an LDA score higher than 2

Next-generation sequencing of bacterial 16S rRNA genes from fecal samples collected at baseline comprised Firmicutes and Bacteroidetes as the two most abundant phyla (Fig. 1a). *Bacteroides, Prevotella, Faecalibacterium*, unclassified genera in *Ruminococcaceae, Lachnospiraceae* and *Rikenellaceae* families, and an unclassified family in *Clostridiales* order were the most abundant genera representing more than 5% of the relative abundance at the baseline of both MFD- and CD (Fig. 1b). There were no significant differences between MFD and CD at baseline or at the end of the study, at phylum or genus taxonomic levels (Fig. 1a, b). However, there was an increased *Prevotella*/*Bacteroides* ratio in MFD group after the dietary intervention, although this difference was not significant (Fig. 1c). These bacteria have previously been described as important for benefits of barley on metabolic markers [19]. There were no significant differences between MFD and CD in α-diversity and no significant clustering of the groups in the PCoA of weighted Unifrac phylogenetic distance metrics (Fig. 1d). In contrast, deeper analysis at species level using the LEfSE biomarker discovery tool revealed that *Prevotella copri* increased significantly in abundance within the MFD group after the dietary intervention compared to the baseline levels (Fig. 2a). LEfSE utilizes a two-step statistical analysis including a nonparametric Kruskal–Wallis sum-rank test to identify bacterial taxa differing significantly in abundance between groups, followed by Linear Discriminant Analysis to estimate the effect size of each differentially abundant feature. A regular *t* test did not detect significant differences in *P. copri* levels (Fig S1). Hence, the effects on *P. copri* are likely borderline significant with LEfSE and must be interpreted with care. Within the control group, no significant differences at species level could be detected, but four genera and one unclassified family of bacteria increased in abundance at the end of the study compared to the baseline level (Fig. 2b).

### Associations between intake of MFD, gut microbiota and cardiometabolic risk markers

**Fig. 3:**
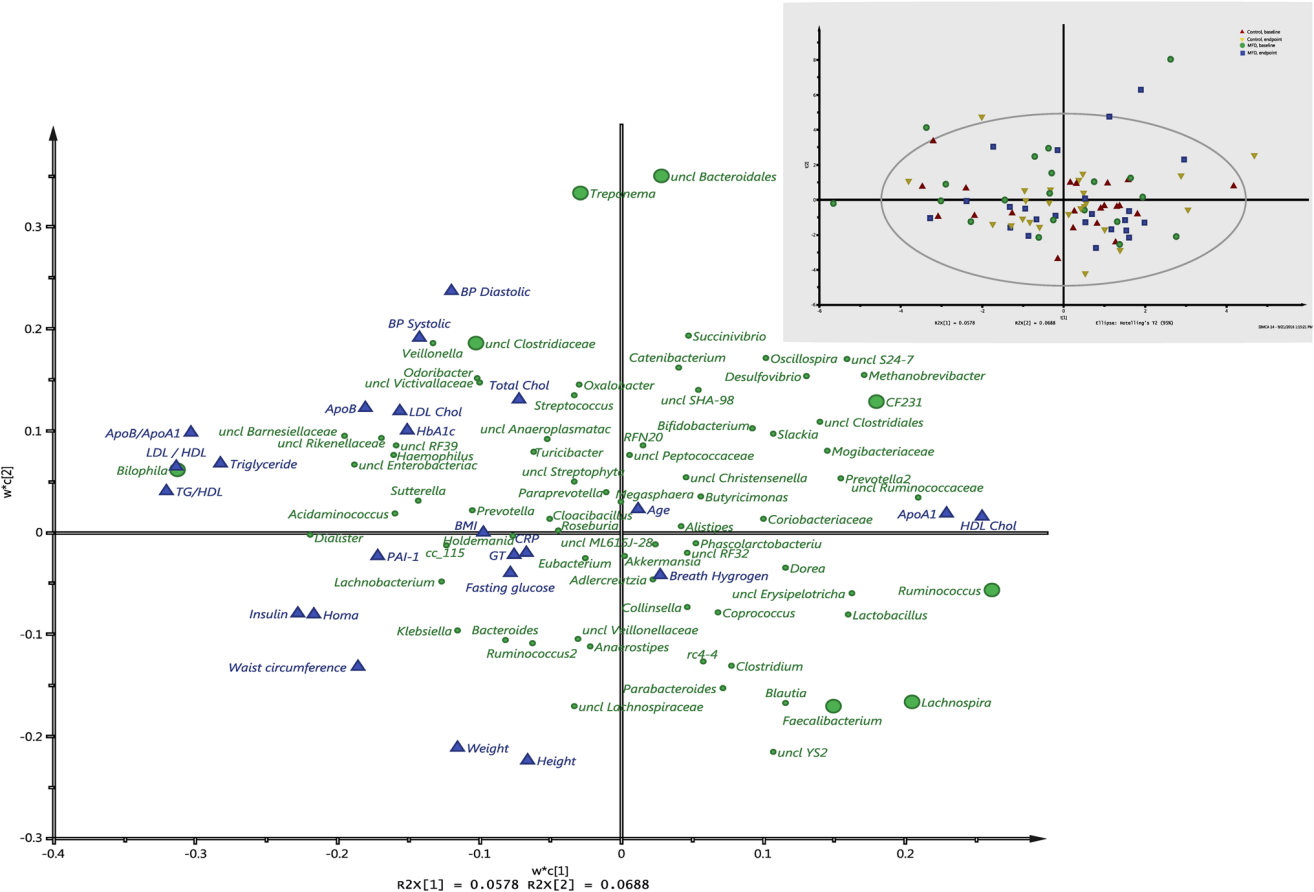
Loading (big panel) and score scatter (small) PLS plots illustrating correlations between gut microbiota at genus level and cardiometabolic risk markers in MFD and control diet groups. Bacterial genera significantly correlated with the risk markers are shown in big green circles. Uncl, unclassified

We next sought to determine whether cardiometabolic risk parameters were associated with bacterial taxa, and found that eight genera were significantly correlated with risk markers (Fig. 3). *Treponema*, an unclassified family in *Bacteroidales* order (adjusted *P* < 0.001) and an unclassified genus in *Clostridaceae* family (adjusted *P* < 0.05) were positively associated with blood pressure, while *Faecalibacterium* (adjusted *P* < 0.05) and *Lachnospira* (adjusted *P* < 0.001) were negatively correlated. Regarding HDL levels, *CF231* (adjusted *P* < 0.001) and *Ruminococcus* (adjusted *P* < 0.05) correlated positively with HDL, while *Bilophila* correlated negatively (adjusted *P* < 0.05). Furthermore, *Bilophila* also correlated positively with LDL/HDL ratio, ApoB/ApoA1 ratio and with TG/HDL ratio (adjusted *P* < 0.001).

## Discussion

The MFD was effective in improving cardiometabolic risk markers and in the present study, we show that it induced minor shifts in the gut microbiota at species level, with increased abundance of *P. copri. Prevotella* has recently been found to be one of the three genera driving enterotypes of the human gut microbiota; *Bacteroides* (Enterotype 1), *Prevotella* (Enterotype 2) *and Ruminococcus* (Enterotype 3) [20]. A long-term diet enriched in carbohydrates has been linked to the *Prevotella* enterotype, while protein and animal fat has been linked to the *Bacteroides* enterotype [21]. *Prevotella*/*Bacteroides* ratio and, particularly, *P. copri* has recently been found to be associated with improved glucose tolerance after intake of barley kernels [22]. In the present study, the MFD resulted in improved fasting glycaemia and insulinaemia, although the effect was not significantly different from that exerted by CD [8].

We found that a number of bacterial genera were associated with the investigated biomarkers of cardiometabolic risk. Of note, *Treponema* correlated positively with blood pressure. This bacterium has been implicated in periodontal disease, a known risk factor for atherosclerosis, and its abundance in the oral cavity has, interestingly, also recently been associated with obesity in humans [23]. Our study shows that the gut abundance of *Treponema* may also be linked to cardiometabolic risk factors such as increased blood pressure. In contrast, *Faecalibacterium* showed a negative association with blood pressure, which may be a reflection of its proven anti-inflammatory capacities [24]. In addition, patients with the metabolic syndrome (MetS) show a reduction in *Faecalibacterium prausnitzii* compared to healthy individuals, which was restored upon a dietary intervention with a Mediterranean-type diet [25]. Three bacterial genera were also found to correlate with blood lipid levels. Certain bacterial species are known to possess bile-salt hydrolase activity, such as some lactobacilli and bifidobacteria species, which may interfere with bile salt activity in the gut and, consequently, bile salt reabsorption and cholesterol synthesis in the liver [26]. In our study, *Ruminococcus* and *CF231* were associated with increased HDL levels, while *Bilophila* appeared to be associated with less favorable blood lipid profiles. *Bilophila wadsworthia* has been implicated in colitis in mice and it increases after high intake of saturated milk-fat through alterations in bile acid profiles [27]. Although the MFD did not significantly alter the gut abundance of these bacteria, our correlation analyses of bacterial taxa with cardiovascular risk markers identified members of the gut microbiota that can be targeted in future dietary interventions to improve cardiometabolic risk markers.

The diet is regarded as a major determinant of gut community structure, but in our study the effects of the MFD induced relatively small changes in the gut microbiota composition compared to the control diet, with significant changes only at the species level. Dietary fiber is the main food component reaching the colon undigested, thus acting as substrate for the microbiota. Although the dietary fiber content was higher in the MFD than in the CD (62 versus 24 g/day, respectively), the dietary fiber content in CD almost reaches the level of dietary fiber intake recommendation of 25–35 g/day in adults [10]. Given that 24 g/day of dietary fiber in CD should be enough to provide substrate for the gut microbiota, appreciable fermentation should take place. The 32% reduction in breath hydrogen, i.e., one of the products from bacterial fermentation, in CD individuals at the endpoint may suggest that some or even most individuals may have included high amounts of dietary fiber in their diets already before the intervention. Further, there was a large variation in the individuals’ microbiota already at the baseline of the study, probably due to variations in their daily dietary fiber sources and intake levels. The period of 8-week intervention may not be enough to induce changes in the gut microbiota towards one direction when the niches are different from the beginning. The large variation of the gut microbiota in these individuals remained at the endpoint of the study, thus possibly reducing the chance of detecting differences due to the diet. Consideration of the daily dietary fiber intake in the inclusion criteria when recruiting subjects for such dietary intervention studies may help to reduce the big gut microbiota variation at baseline. Also, a larger sample size could have helped to clarify further whether the MFD affected also other bacteria than those reported in the present study. In addition, the control diet was a “healthy” diet, formulated according to the Nordic Nutrition Recommendations, while a more extreme, unhealthy control diet may have resulted in larger differences between groups.

In conclusion, the MFD did not alter the gut microbiota at phylum or genus level. At species level, *P. copri* was identified by the biomarker discovery tool LEfSE as discriminant for the MFD and its role in improvement of risk markers should be investigated further. Furthermore, gut abundance of several bacteria correlated with blood pressure, including *Faecalibacterium* that correlated negatively with blood pressure and *Treponema* that correlated positively, while *Bilophila* appeared to associate with an unfavorable blood lipid profile. Thus, the results can be used to further optimize health effects of the MFD, by addressing bacteria associating with cardiometabolic risk markers. Taken together, results from the present study may be used in the further development of effective dietary concepts capable of reducing cardiometabolic risk markers in humans through a targeted modulation of the gut microbial community.

## Electronic supplementary material

Below is the link to the electronic supplementary material.

Supplementary material 1 (DOCX 17 KB)

Figure S1. Relative abundance of *P. copri* in MFD and control diet groups. Data shown are mean and standard deviation. (TIF 173 KB)

Figure S2. Loading (big panel) and score scatter (small) PLS plots illustrating correlations between gut microbiota from de novo OTU picking dataset and cardiometabolic risk markers in MFD and control diet groups. Bacterial genera significantly correlated with the risk markers are shown in big green squares. Uncl, unclassified. (TIF 4374 KB)
