# Supplementary material for: Improvement in cardiometabolic risk markers following a multifunctional diet is associated with gut microbial taxa in healthy overweight and obese subjects
Source: Eur J Nutr. 2017 Nov 2;57(8):2927–36. doi: 10.1007/s00394-017-1563-3 (PMC6267413; doi:10.1007/s00394-017-1563-3)
Supplement: Supplementary file 1 — Supplementary material 1 (DOCX 17 KB) [file 394_2017_1563_MOESM1_ESM.docx]

**Supplementary material**

Representative 1-day menus for the multifunctional diet and the control diet.

| **MFD** | **CD** |
| --- | --- |
| *Breakfast* |  |
| Soy-based yoghurt | Yoghurt/sour milk (1.5 % fat,  unsweetened) |
| Oat-fiber muesly | Corn flakes |
| Rye/oat muesly | Apple purée |
| Blueberries |  |
| Cinnamon |  |
| *Snack* |  |
| Guar gum bread | White wheat bread |
| Stanol-containing margarine Margarine | Cheese (28% fat) |
|  | Cucumber |
| *Lunch* |  |
| Rye-barley bread | White wheat bread |
| Guar gum bread | Wheat crisp bread |
| Rye crisp bread | Margarine (butter-oil blend) |
| Stanol-containing margarine | Cheese (28% fat) |
| Canned mackerel in tomato sauce | Smoked sausage (30% fat) |
| Lean ham (max. 3% fat) | Mixed greens and vegetables |
| Mixed greens and vegetables | Orange marmalade |
| Cheese (17% fat) |  |
| *Dinner* |  |
| Lean beef | Blended (pork/beef) mince |
| Parsnip | Margarine (butter-oil blend) |
| Potatoes | Carrot |
| Onion  Whey protein | Canned tomatoes |
| Canned soybeans | Sweet corn (frozen) |
| Rapeseed oil | Pasta |
| Carrot | Parmesan cheese |
| Lettuce | Lettuce |
| Salad dressing |  |
| *Snack* |  |
| Almonds (with peel) | Cookies or sweet wheatbun |
| Oat-based drink | Crisp rolls (wheat flour) |
| Blueberry purée | Margarine (butter-oil blend) |
| Mixed fruits (min. 3/day) | Mixed fruits (min. 3/day) |

**Additional analyses of the gut microbiome: PICRUSt, *de novo* OTU picking and identification of responders**

The functional capacity of the gut microbiome was inferred from the 16S rRNA gene sequencing data using PICRUSt analysis, but no significant differences between MFD and CD were observed. Using the broader *de novo* OTU picking method, which aligns sequences against each other without the use of a reference database (thus not discarding sequences not matching the database), we could compare our results with the closed-reference OTU picking method, and identified a total number of 159,249 OTU:s, while the closed-reference method identified 5,756 OTUs. Applying the *de novo* dataset to the PLS correlation analysis revealed largely the same clusters, where the MFD associated negatively with total cholesterol, LDL and LDL/HDL ratio, and positively with *Prevotella* genus. However, none of these associations reached statistical significance (Fig. S2).
