# Supplementary figures and images for: Improvement in cardiometabolic risk markers following a multifunctional diet is associated with gut microbial taxa in healthy overweight and obese subjects
Source: Eur J Nutr. 2017 Nov 2;57(8):2927–36. doi: 10.1007/s00394-017-1563-3 (PMC6267413; doi:10.1007/s00394-017-1563-3)

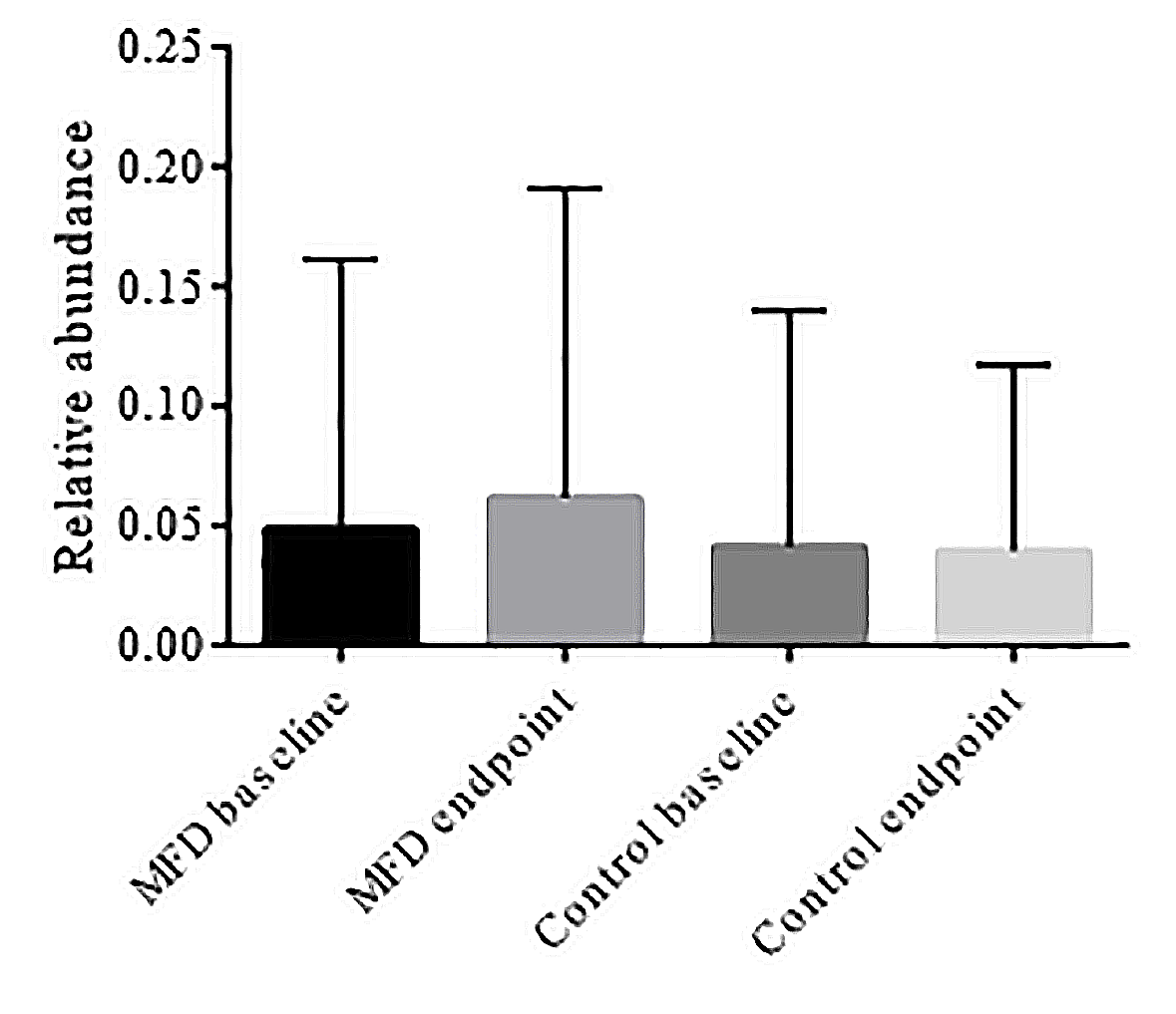

Supplement: Supplementary file 2 — Figure S1. Relative abundance of P. copri in MFD and control diet groups. Data shown are mean and standard deviation. (TIF 173 KB) [file 394_2017_1563_MOESM2_ESM.tif]

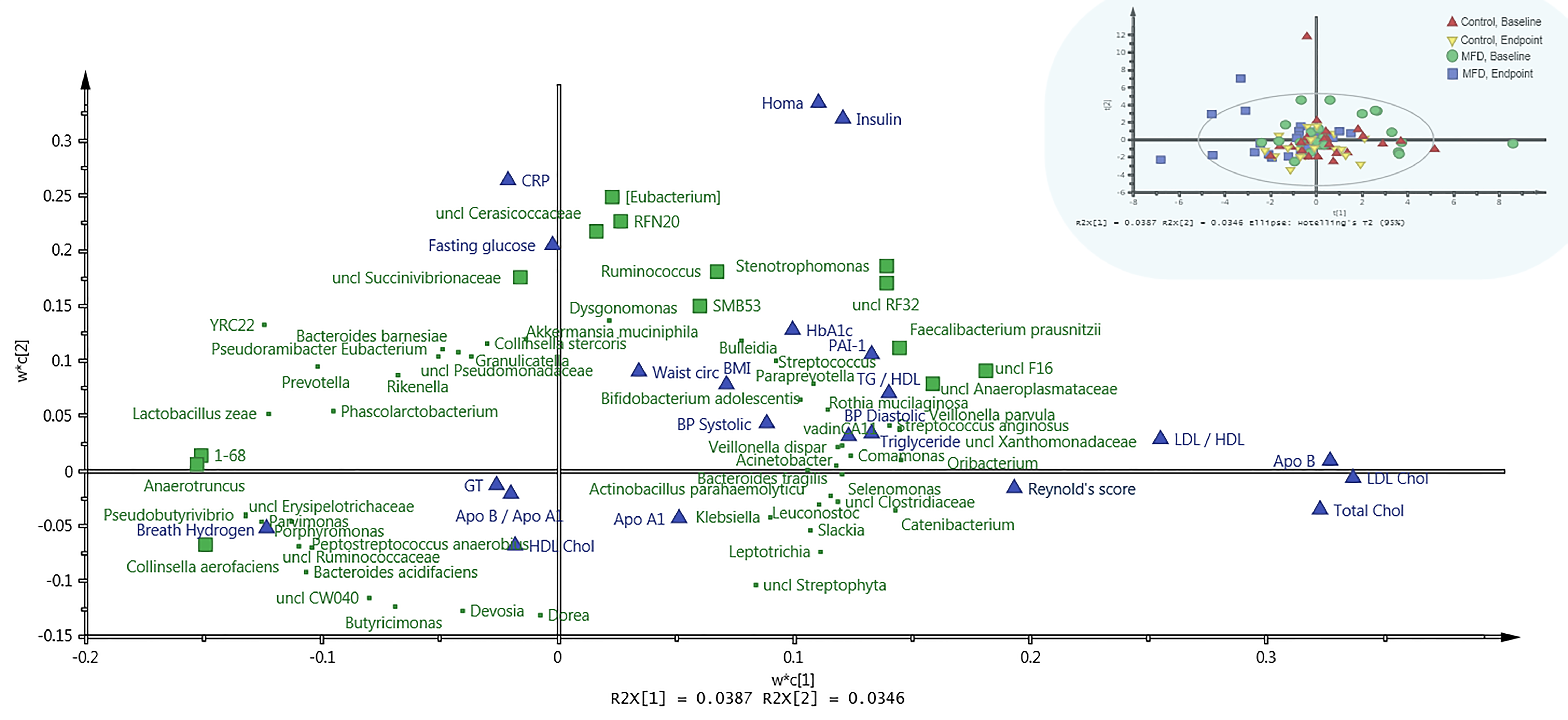

Supplement: Supplementary file 3 — Figure S2. Loading (big panel) and score scatter (small) PLS plots illustrating correlations between gut microbiota from de novo OTU picking dataset and cardiometabolic risk markers in MFD and control diet groups. Bacterial genera significantly correlated with the risk markers are shown in big green squares. Uncl, unclassified. (TIF 4374 KB) [file 394_2017_1563_MOESM3_ESM.tif]
